# Illicit and pharmaceutical drug consumption estimated via wastewater analysis. Part B: Placing back-calculations in a formal statistical framework

**DOI:** 10.1016/j.scitotenv.2014.02.101

**Published:** 2014-07-15

**Authors:** Hayley E. Jones, Matthew Hickman, Barbara Kasprzyk-Hordern, Nicky J. Welton, David R. Baker, A.E. Ades

**Affiliations:** a School of Social and Community Medicine, University of Bristol, Canynge Hall, 39 Whatley Road, Bristol BS8 2PS, UK; b Department of Chemistry, University of Bath, Faculty of Science, Bath BA2 7AY, UK; c Mass Spectrometry Business Unit, Shimadzu, Wharfside, Manchester M17 1GP, UK

**Keywords:** Sewage epidemiology, Monte Carlo simulation, Uncertainty propagation, Bayesian modelling, Illicit drugs

## Abstract

Concentrations of metabolites of illicit drugs in sewage water can be measured with great accuracy and precision, thanks to the development of sensitive and robust analytical methods. Based on assumptions about factors including the excretion profile of the parent drug, routes of administration and the number of individuals using the wastewater system, the level of consumption of a drug can be estimated from such measured concentrations. When presenting results from these ‘back-calculations’, the multiple sources of uncertainty are often discussed, but are not usually explicitly taken into account in the estimation process. In this paper we demonstrate how these calculations can be placed in a more formal statistical framework by assuming a distribution for each parameter involved, based on a review of the evidence underpinning it. Using a Monte Carlo simulations approach, it is then straightforward to propagate uncertainty in each parameter through the back-calculations, producing a distribution for instead of a single estimate of daily or average consumption. This can be summarised for example by a median and credible interval. To demonstrate this approach, we estimate cocaine consumption in a large urban UK population, using measured concentrations of two of its metabolites, benzoylecgonine and norbenzoylecgonine. We also demonstrate a more sophisticated analysis, implemented within a Bayesian statistical framework using Markov chain Monte Carlo simulation. Our model allows the two metabolites to simultaneously inform estimates of daily cocaine consumption and explicitly allows for variability between days. After accounting for this variability, the resulting credible interval for average daily consumption is appropriately wider, representing additional uncertainty. We discuss possibilities for extensions to the model, and whether analysis of wastewater samples has potential to contribute to a prevalence model for illicit drug use.

## Highlights

• Analysis of wastewater allows estimation of illicit drug consumption.
• However, it is crucial to formally acknowledge the many sources of uncertainty.
• The simple and flexible Monte Carlo simulation approach allows this.
• There are many software options: we provide an Excel spreadsheet and R code.
• Bayesian modelling using Markov chain Monte Carlo allows interesting extensions.

## 1 Introduction

The analysis of communal sewage water entering wastewater treatment plants (WWTPs) offers potential for enhancing our knowledge of illicit drug consumption (Daughton, 2001; Frost et al., 2008; van Nuijs et al., 2011a; Zuccato et al., 2008). State-of-the-art sensitive and robust analytical methods mean that concentrations of drug target residues (DTRs), such as metabolites of an illicit drug, in wastewater can be measured with great accuracy and precision (Baker and Kasprzyk-Hordern, 2011b; Castiglioni et al., 2013). In what has been termed the ‘sewage epidemiology’ approach, consumption of the parent drug is ‘back-calculated’ from these measured DTR concentrations (Zuccato et al., 2005). For example, our sister paper, entitled ‘Illicit and pharmaceutical drug consumption estimated via wastewater analysis. Part A: Chemical analysis and drug use estimates’ (Baker et al.,2014--in this issue), describes a study of a large UK population.

The results of these calculations are of course only estimates of illicit drug use, subject to many sources of uncertainty. As the field develops, it is important that this is properly addressed. Key variables include the size of the population served by the WWTP and the percentage of a dose of the parent drug that is excreted as the DTR. In addition, there is evidence that this percentage varies according to the route of administration of the parent drug (Khan and Nicell, 2011). Hence data are also required on the distribution of routes of administration across the population. All parameters informed by data are subject to sampling variation.

Usually, as in Baker et al. (2014--in this issue), only the analytical uncertainty in the measurement of DTR concentrations in a wastewater sample has been explicitly taken into account. Since this uncertainty is generally very small, back-calculated drug consumption estimates often incorrectly appear to be very precise. To avoid over-interpretation of the estimates, it is highly desirable to present credible intervals around them, accounting for as many additional sources of uncertainty as possible. Recently, Lai et al. (2011) and Mathieu et al. (2011) have attempted to propagate uncertainty in multiple parameters simultaneously through the back-calculations. However, as we will discuss below, their approach has limited applicability.

In this paper we propose Monte Carlo simulation as a simple and more general approach to accounting for multiple sources of uncertainty in sewage epidemiology back-calculations. The approach involves specifying a probability distribution, based on ‘all available evidence’, for each of the parameters involved. The specified distributions are repeatedly sampled from at random, and the back-calculations performed for each set of simulated values. The end result is a simulated distribution for consumption of the parent drug, from which summary statistics can be presented which appropriately reflect the uncertainties. Monte Carlo simulation has been routinely used to propagate uncertainty in models in the physical and social sciences since use of computers became widespread (Metropolis and Ulam, 1949). It also has a key role in decision making (Critchfield and Willard, 1986; Doubilet et al., 1985), as it provides a simple way of estimating expectations under uncertainty in non-linear models. To demonstrate its application to wastewater analysis, we use data from the Part A paper (Baker et al.,2014--in this issue) to ‘back-calculate’ cocaine consumption based on concentrations of the metabolites benzoylecgonine and norbenzoylecgonine (Section 3).

A further possibility, which we illustrate in Section 4, is simulation from a Bayesian joint posterior distribution using Markov chain Monte Carlo (Gilks et al., 1996). This has the advantage of combining simulation with statistical estimation of parameters from multiple data sources. This approach – sometimes called ‘comprehensive decision analysis’ – has been popular in decision sciences for over 30 years (Parmigiani, 2002; Samsa et al., 1999; Spiegelhalter et al., 1999). For wastewater analysis, it opens up possibilities for many more sophisticated statistical analyses, such as modelling variability over time or allowing consumption of a drug to be simultaneously informed by concentrations of multiple DTRs.

## 2 Background: ‘back-calculation’ of drug consumption using DTR concentrations

Baker et al. (2014--in this issue) present estimates of drug and pharmaceutical consumption in a large (estimated 3.4 million) urban UK population. They used the following modified versions of formulae introduced by Zuccato et al. (2005) to estimate per capita consumption from measured DTR concentrations:*Load of DTR in grams grammes per day*

(1) $$\mathrm{Load}=\frac{\mathrm{Concentration}\times \mathrm{Flow}}{1000}\times \left(\frac{100}{100+\mathrm{Stability}}\right)\times \left(\frac{100}{100-\mathrm{Sorption}}\right)$$

where*Concentration* = DTR concentration in wastewater influent (ng/l),*Flow* = volume of flow to the wastewater influent over a 24 hour period (millions of litres/day),*Stability* = percentage change in concentration of the DTR in wastewater in the conditions (time, pH and temperature) relevant to the study, and *Sorption* = percentage sorption of the DTR to suspended particulate matter (SPM) in wastewater.

*Estimated drug consumption in mg/day per 1000 people*

(2) $$\mathrm{Consumption}=\left(\frac{\mathrm{Load}}{\mathrm{Population}\times \mathrm{Excretion}}\right)\left(\frac{MW_{\mathrm{Par}}}{MW_{\mathrm{DTR}}}\right)-\mathrm{OS}$$

where *Excretion* = proportion of a dose of the parent drug excreted as the DTR, *MW_Par_* = molecular weight of the parent compound, *MW_DTR_* = molecular weight of the DTR, *Population* = size of the population served by the WWTP (millions), and *OS* = the amount of the DTR present in wastewater due to sources other than consumption of the parent compound (e.g. hospital or prescription usage).

For drugs such as cocaine that are administered using multiple routes by different users, the typical metabolism profile of the drug will likely vary according to this. As such, *Excretion* should be estimated as an average over the different routes (Khan and Nicell, 2011):*Proportion of a dose of the parent drug excreted as the DTR*

(3) $$\mathrm{Excretion}=\Sigma_{R}\left[\left(\mathrm{proportion}\;\mathrm{of}\;\mathrm{all}\;\mathrm{parent}\;\mathrm{drug}\;\mathrm{mass}\;\mathrm{that}\;\mathrm{is}\;\mathrm{administered}\;\mathrm{by}\;\mathrm{route}\;R\right)\times \left(\mathrm{proportion}\;\mathrm{of}\;a\;\mathrm{dose}\;\mathrm{of}\;\mathrm{the}\;\mathrm{parent}\;\mathrm{drug}\;\mathrm{excreted}\;\mathrm{as}\;\mathrm{the}\;\mathrm{DTR}\;\mathrm{following}\;\mathrm{administration}\;\mathrm{by}\;\mathrm{route}\;R\right)\right]$$

Except for the molecular weights, there is uncertainty about all of these parameters. Failure to take these uncertainties into account is likely to lead to over-interpretation of the results.

When uncertainty about the individual parameter values involved in the back-calculations has been quantified, it has generally been expressed as relative standard deviations (SD) (Castiglioni et al., 2013). The RSD is defined as the standard deviation divided by the absolute value of the parameter estimate. We note that there is ambiguity here in the meaning of ‘standard deviation’. Consider, for example, the *Excretion* factors in Eq. (3). Clearly the metabolism profile of a drug will vary across individuals, according for example to genetic factors. This variability is quantified by the standard deviation. But for valid inference on consumption by a large population, only the *average* excretion profile across the population of users need be well estimated. The standard deviation of a parameter estimate is usually called the ‘standard error’ (SE) in statistics. The SE is the more appropriate measurement of uncertainty about the parameter used in the back-calculation. It can be reduced by the collection of new data, whereas the standard deviation (SD) cannot. In the simple case where a parameter has been estimated by the arithmetic mean of n data points, the SE is calculated as $\mathrm{SD}/\sqrt{n}$. When the parameter estimate is a weighted average of estimates across multiple studies, then it is the standard error of the pooled estimate that we generally require.

If the formulae for estimating consumption were linear on the log scale, then the square of the RSD of the estimate of consumption could be approximated by the sum of squares of the RSDs of each individual parameter estimate (Lai et al., 2011; see also Mathieu et al. 2011 who used this approach to quantify uncertainty in estimated loads). This may have been a reasonable approach for early back-calculations, when *Stability*, *Sorption* and *OS* were not accounted for and *Excretion* was estimated by a single value rather than by averaging across routes of administration (Zuccato et al., 2005). But clearly consumption as estimated by Eqs. (1)–(3) above is no longer a linear function of the underlying parameters on the log scale. In addition, daily estimates of consumption are often averaged over multiple days. As the calculations rely on increasing numbers of parameters, and the function of parameters being estimated becomes more complex, a more general approach to accounting for uncertainty is required.

## 3 Propagation of uncertainty using Monte Carlo simulation

Monte Carlo simulation offers a simple and highly flexible alternative to the approach of Lai et al. (2011). The first stage is to characterise each parameter in terms of a statistical distribution that reflects the uncertainty about its value, based ideally on data, or on expert opinion if no data are available. The choice of distribution should reflect the target properties of the parameter and the distribution of any data informing it (see e.g. Hunink, 2001, p351). For most parameters, uncertainty can be represented using a normal distribution with mean equal to the parameter estimate and standard deviation equal to the SE of that estimate. In particular, if the parameter estimate is a sample mean then the normal distribution can be considered appropriate by the Central Limit Theorem, assuming that the sample is reasonably large. The distribution of a proportion must of course lie entirely between 0 and 1. Therefore a beta distribution, or a normal distribution on a logit scale, would be preferred, since either would enforce this constraint.

Values for the individual parameters are then simulated at random from each of the assumed distributions, and the back-calculation (Eqs. (1)–(3)) performed using these simulated values. This is repeated many times, to obtain a simulated distribution for consumption of the parent drug. The mean or median of these values serves as a point estimate, while the 2.5th and 97.5th percentiles are a 95% credible interval (Cr-I). This indicates the range in which there is a 95% probability that the true value lies (assuming the distributions are accurate). The standard deviation of the simulated values represents our uncertainty about the estimate of consumption, and can be interpreted as the SE of the estimate. Note that these simulation-based results are themselves subject to a degree of random sampling error, known as Monte Carlo error. This is easily reduced by increasing the number of simulations.

As a demonstration, we will estimate cocaine consumption using measured concentrations of the two metabolites benzoylecgonine and norbenzoylecgonine, as presented by Baker et al. (2014--in this issue). We show the daily measured concentrations of these two DTRs over the sampling period in Table 1. In brief, a 24 hour composite sample of wastewater was taken on each of seven days. Each daily sample was split into two and analysed separately in order to assess analytical uncertainty in measurement of metabolite concentration. The values reported in Table 1 are mean concentrations across the two sub-samples for each of the seven days, along with SEs of these means. Refer to Baker et al. (2014--in this issue) for details of the sampling and analytical methodology. We note that concentrations of these two cocaine metabolites across days are very highly correlated (sample correlation between mean daily concentrations = 0.95).

**Fig. 1:**
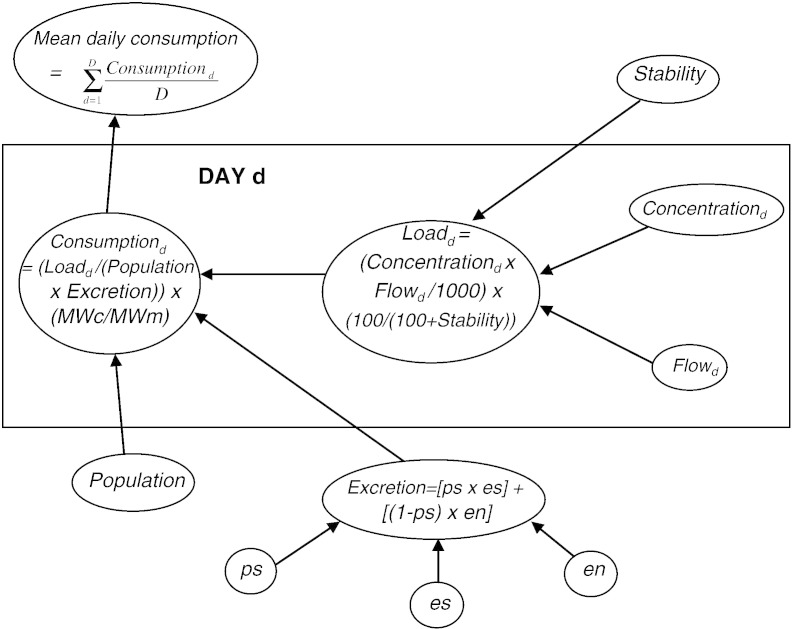
Directed acyclic graph for ‘back-calculation’ approach (single metabolite). Consumption_d_ = cocaine consumption per 1000 people on day d (mg); Load_d_ = load of metabolite entering the wastewater system on day d (grammes); Population = size of the population served by the WWTP (millions); Concentration_d_ = concentration of metabolite in sample of wastewater on day d (ng/l); Flow_d_ = total volume of flow to the wastewater influent on day d (millions of litres); Stability = percentage change in concentration of metabolite in wastewater in the conditions relevant to the study; Excretion = proportion of consumed cocaine that is excreted as the metabolite; MWm = molecular weight of metabolite; MWc = molecular weight of cocaine; ps = proportion of all cocaine mass that is smoked as free base, es = proportion of a dose of cocaine smoked as free base that is excreted as the metabolite; en = proportion of a dose of cocaine consumed by nasal insufflation that is excreted as the metabolite.

For this application, we will ignore the *Sorption* and *OS* factors in Eqs. (1)–(2), since there is negligible sorption of benzoylecgonine and norbenzoylecgonine to SPM (Baker et al.,2014--in this issue; Baker and Kasprzyk-Hordern, 2011c) and other sources of these two metabolites were assumed negligible. For Eq. (3) we assume that all cocaine consumed by this population was administered by smoking or nasal insufflation. The simplified equations for one metabolite are shown as a ‘directed acyclic graph’ (DAG) in Fig. 1. The arrows indicate the direction of the flow of information between parameters. We estimate per capita cocaine consumption for each day separately, and also estimate average consumption across the seven days of sampling. In the DAG, everything within the rectangle relates to a specific day (d = 1,…,7) while parameters outside of the rectangle are assumed common across all days.

We use the same parameter estimates as Baker et al. (2014--in this issue). Tables 1 and 2 show the distribution assumed for each parameter, and brief descriptions of how the estimates and SEs were arrived at. For the *Excretion* proportions relating to benzoylecgonine, results from multiple small pharmacokinetic studies were available, a review of these being presented by Khan and Nicell (2011) (see also Castiglioni et al. (2013)). We calculated weighted averages across these results using a simple technique known as meta-analysis (see, for example, Borenstein, 2009). Results from multiple studies are also available for *Stability* (Castiglioni et al., 2013; van Nuijs et al., 2012), and could be averaged across in the same way. However, we deemed it more appropriate to use the results from Baker and Kasprzyk-Hordern (2011a), since we found no other results relating to the same pH level, temperature and storage time. The population size estimate was provided by wastewater personnel and we unfortunately lacked information on how precise this might be. In this situation, it is better to set the SE to allow for a reasonable degree of uncertainty rather than to pretend that the parameter value was known exactly. We therefore assumed a value for the SE such that the limits of a 95% confidence interval accommodated a 10% relative error in either direction (see Table 2). We assumed a beta distribution for all proportions, in order to constrain these to within the range [0,1]. The parameters of the beta distribution can be estimated to correspond to the required means and SEs (Table 2). We assumed a normal distribution for all other parameters.

It is straightforward to implement the Monte Carlo simulation approach in many software packages. Since the majority of readers will be familiar with Excel, we provide an Excel spreadsheet that performs the cocaine consumption estimation based on benzoylecgonine concentrations as an online appendix. However, models written in Excel lack transparency, and it is easy to make errors. In practice, we therefore encourage use of statistical software such as R, SPlus or Stata. Code for performing the same analysis using R is therefore also provided in the online materials.

**Fig. 2:**
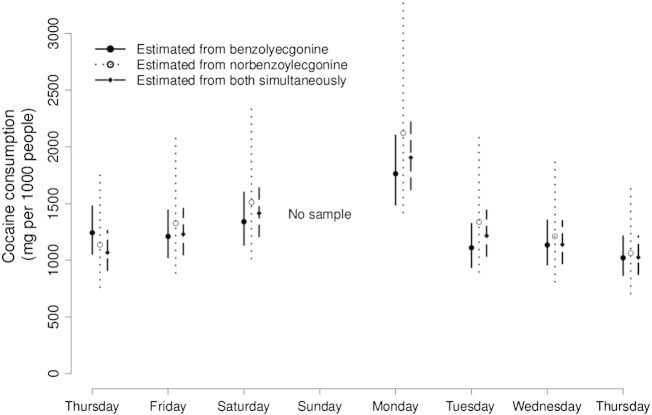
Estimated daily cocaine consumption in a large UK city, based on measured concentrations of benzoylecgonine and norbenzoylecgonine. Estimates with 95% Cr-Is based on the ‘back-calculation’ approach applied to each metabolite separately, with propagation of multiple sources of uncertainty using Monte Carlo simulation (Section 3). The third estimate and 95% Cr-I presented for each day is based on a fully Bayesian analysis of both metabolites simultaneously, with a common distribution assumed for true daily consumptions (Section 4). The standard deviation of consumption across days on the log scale (τ) was estimated to be 0.25 (95% Cr-I 0.14–0.57).

In Fig. 2 we show daily estimates of cocaine consumption based on independent analyses of the benzoylecgonine and norbenzoylecgonine data, with the 95% Cr-Is reflecting all of the uncertainties described in Tables 1 and 2 (the third set of estimates displayed will be explained in Section 4). As is clear from the figure, the estimates based on the two metabolites are very highly correlated, reflecting the high observed correlation in measured concentrations. For 6 of the 7 days of sampling, cocaine consumption was estimated to be slightly higher based on measured norbenzoylecgonine than benzoylecgonine concentrations. The estimated mean daily cocaine consumption based on analysis of the benzoylecgonine data was 1260 mg per 1000 people (95% Cr-I 1074 to 1494), compared with 1387 mg per 1000 people (95% Cr-I 932 to 2164) based on the norbenzoylecgonine data. The overlapping 95% credible intervals, both for the mean and daily consumption estimates, demonstrate that the two sets of results are however consistent with each other.

The credible intervals based on norbenzoylecgonine concentrations are considerably wider than those based on benzoylecgonine. This reflects the fact that norbenzoylecgonine is a much more minor metabolite of cocaine, with only about 1% of a typical dose being excreted as it, compared with about 30% as benzoylecgonine. This means that any uncertainties regarding excretion factors are magnified to a greater extent in the back-calculations (that is, the relative uncertainty is larger).

## 4 Extended analysis using a Bayesian Markov chain Monte Carlo simulation approach

A natural extension to the approach described in Section 3 is to combine parameter estimation and uncertainty propagation in a single step, which can be achieved within a Bayesian statistical framework computed using Markov chain Monte Carlo (MCMC) simulation (Gilks et al., 1996). Like the Monte Carlo approach, MCMC involves repeatedly simulating values for parameters and functions of parameters, and making inferences based on summary statistics of simulated distributions. The distinction is that while in Monte Carlo simulation the distribution of each parameter is completely pre-specified, in Bayesian MCMC a ‘prior’ distribution is specified for each parameter, which is updated to form a ‘posterior’ distribution based on the addition of relevant new data to the model. Moving to full MCMC estimation, which can be implemented using the WinBUGS software (Lunn et al., 2000, 2012), opens up possibilities for a wide range of analyses. We will use MCMC to extend our analysis of the UK cocaine example, allowing measured concentrations of the two metabolites to simultaneously inform estimates of daily consumption, and addressing day to day variability.

**Fig. 3:**
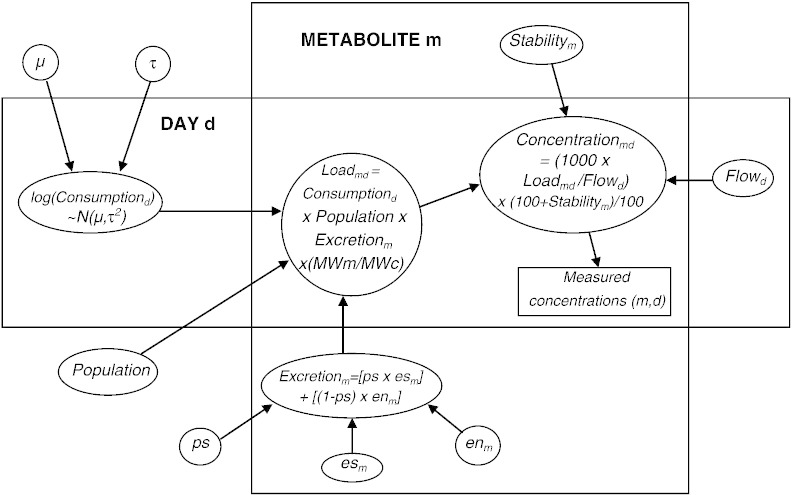
Directed acyclic graph for Bayesian analysis of 2 metabolites simultaneously. Consumption_d_ = cocaine consumption per 1000 people on day d (mg); Load_md_ = load of metabolite m entering the wastewater system on day d (grammes); Population = size of the population served by the WWTP (millions); Concentration_md_ = concentration of metabolite m in sample of wastewater on day d (ng/l); Flow_d_ = total volume of flow to the wastewater influent on day d (millions of litres); Stability_m_ = percentage change in concentration of metabolite m in wastewater in the conditions relevant to the study; Excretion_m_ = proportion of consumed cocaine that is excreted as metabolite m; MWm = molecular weight of metabolite m; MWc = molecular weight of cocaine; ps = proportion of all cocaine mass that is smoked as free base, es_m_ = proportion of a dose of cocaine smoked as free base that is excreted as metabolite m; en_m_ = proportion of a dose of cocaine taken by nasal insufflation that is excreted as metabolite m.

Conceptually, the Bayesian approach involves reconsidering the direction of relationships between the parameters described in Fig. 1. In reality of course it is the amount of consumption of the parent drug that, along with other parameters, determines loads and concentrations of the DTRs — not the other way around. In Fig. 3 the ‘back-calculations’ (Eqs. (1) and (2)) are rewritten in the more natural ‘forwards’ formulation in a new DAG, representing our extended analysis. The measured concentrations of each metabolite on each day are now viewed as data, usually represented by a rectangle in a DAG. The data are realisations of functions of multiple parameters, some of which we know about and others (consumption of the parent drug) that we don't. For the parameters that we know about (excretion rates, population size, stability and flow), we can assume the same distributions as in Section 3. In Bayesian modelling we refer to these as ‘informative’ prior distributions. In contrast, daily consumption is unknown and is therefore assigned a vague or ‘uninformative’ prior distribution. Consequently the posterior distribution of consumption on each day is driven by the observed data.

A simplistic relationship between measurements on multiple DTRs is illustrated in Fig. 3: here, daily cocaine consumption by the population, along with excretion factors, drives loads of each metabolite (m = 1,2) in the wastewater. In addition, a hierarchical model structure (see e.g. Goldstein, 2011) is assumed for daily consumption by the population. That is, the true amount of cocaine consumption is allowed to vary across days, but these repeated measures are assumed to be related through a common distribution. Specifically, we assumed a common normal distribution for the logarithm of daily per capita cocaine consumption, with mean μ and standard deviation τ. We chose to assume a normal distribution on the log scale rather than the natural scale because estimates of cocaine consumption based on the Monte Carlo simulation approach were clearly right skewed. Estimation of average daily consumption from this model, rather than by simply taking the arithmetic mean across simulations (Section 3), accounts for the fact that there is more variation between days than can be explained by measurement error.

We used WinBUGS to fit the model described by Fig. 3. Vague prior distributions were assumed for the parameters μ and τ. Posterior estimates (medians) and 95% Cr-Is from this analysis are displayed in Fig. 2. It can be seen that estimates of daily cocaine consumption are largely driven by measured concentrations of benzoylecgonine. Incorporating measurements of the more minor metabolite, norbenzoylecgonine, had relatively little effect. Our model did not allow for correlations due to the two metabolites having been measured in the same samples, or the fact that the excretion factors for these metabolites were informed by some of the same small pharmacokinetic studies. It is possible to allow for complex correlation structures within the modelling approach (Ades and Lu, 2003), but we considered this beyond the scope of our demonstration. We note however that accounting for these sources of correlation would lead to the norbenzoylecgonine measurements having even *less* of an effect, since the amount of ‘extra’ information in the norbenzoylecgonine data would appropriately be considered less. This raises questions as to whether it is worthwhile monitoring additional minor metabolites of a parent drug of interest.

Across the seven days of sampling, the estimated mean daily cocaine consumption from the extended model was 1260 mg (95% Cr-I 960–1662) per 1000 people. Note that this estimate is actually identical to that from the simple Monte Carlo simulation analysis of the benzoylecgonine data alone (Section 3). However, the credible interval is wider, since the new analysis appropriately takes into account the evidence that cocaine consumption varies between days.

Since weekend peaks in consumption of many drugs (including cocaine) are often observed (van Nuijs et al., 2011b), most likely due to increased recreational drug use, it seems appropriate to incorporate a ‘weekend effect’ into the model rather than assuming that consumption on all days simply varies randomly around some mean. For example, we might assume a mean of μ for consumption on the log scale on weekdays, and of μ + δ on weekends. Variability around these means could be assumed either constant across all days or different on weekends. It would be straightforward to extend the WinBUGS model in this way; however longer periods of data than were available for the UK study would clearly be required for reliable estimation of the weekend effect.

Another natural extension to the model would be to allow some of the other parameters in Fig. 3 to be informed by multiple sources of data. For example, rather than placing an informative prior distribution on an excretion rate, based on first performing a meta-analysis of data from multiple pharmacokinetic studies, we could perform the meta-analysis directly within the Bayesian model. This would involve reading the multiple estimates of excretion factors in as additional data (adding additional rectangles representing data to the DAG). In practice this would make little difference to the results, since standard formulae exist for quantifying the uncertainty associated with a pooled estimate from a meta-analysis. However, for some parameters the multiple relevant sources of information will have a more complex relationship between them. For example, the relationship between stability data from multiple studies (Castiglioni et al., 2013; van Nuijs et al., 2012), relating to different pH levels, temperatures and time windows, could be explicitly modelled in order to gain a better estimate of stability in the conditions of interest. Drug consumption modality could potentially be informed simultaneously by population surveys, emergency room data and drug treatment modality, in addition to police seizure data. However, the parameters needed for the analysis are the proportions of cocaine *mass* administered by different routes, not the proportions of *users* reporting different modalities. Therefore the relationship between the two would need to be formally described, involving additional parameters relating to the average daily dosage of different types of user. To combine over such data sources, with appropriate propagation of uncertainty, we would recommend the Bayesian modelling approach.

## 5 Uncertainty beyond statistical sampling error

We have demonstrated how uncertainty due to random sampling error in each parameter estimate can be accounted for. However, it is crucial to recognise that there is also potential for systematic error (bias) in each parameter estimate. Since the amount of bias is unknown, or not known precisely, this leads to additional uncertainty (Greenland, 2005) which is not reflected in the credible intervals we have presented.

For example, the estimate of the population size provided to us by WWTP personnel is almost certainly an estimate of the size of the residential population, which might be quite different from the size of the population using the sewage system. The latter could perhaps vary considerably even within a week, due to travel into and out of the city for work, tourism and leisure purposes. In future studies it is desirable to use hydrochemical parameters (Castiglioni et al., 2013) and/or urinary biomarkers (Daughton, 2012) for more accurate estimation of the population size, preferably providing estimates specific to each day.

Another potential source of bias is in the excretion rates used. Although these appear quite precise from the utilised standard errors in Table 2 (low random error), they could however be biased estimates of the true values (potential systematic error). Neither the individuals studied nor the conditions (e.g. dosage) in the small pharmacokinetic studies carried out to date are necessarily representative of the population of interest. Improved pharmacokinetic information therefore remains a priority for validity of sewage epidemiology back-calculations.

We included only two routes of administration of cocaine in our demonstrative analysis: smoking and nasal insufflation. The proportion of cocaine that is smoked was estimated based on the proportion of all cocaine seized that is crack cocaine, according to data reported by UK police (Coleman, 2012). However, this might not represent the truth well, since seized cocaine is not a random sample of all consumed cocaine. The proportion might also vary geographically and over time. In addition, both cocaine and crack can also be injected. The proportion of injectors is likely to be very small in our population of interest, but it will be important in future studies, particularly in some populations, to account for injecting. With three or more routes of administration, a Dirichlet distribution for the set of proportions would be appropriate, since this automatically constrains their sum to equal one (Briggs et al., 2003). The incorporation of multiple sources of data on drug modality would be desirable, the potential for which we have noted in Section 4.

In this study, 24 hour composite time-proportional samples were collected, involving sample collection every 15 min. To avoid bias in estimated loads, it would be preferable to use flow- or volume-proportional sampling modes (Castiglioni et al., 2013). Bias in estimated loads could also arise from catchment-related characteristics including exfiltration and layout of the WWTP and sewer catchments. For further discussion refer to Castiglioni et al. (2013) and Ort et al. (2010).

We have estimated daily cocaine consumption over seven days of sampling in 2011, and the average consumption over these 7 days. However, only one of the days was on a weekend (there was no sample on Sunday). Since we would expect a weekend peak in drug consumption, the average consumption over these seven days is likely to be a slight underestimate of the consumption over a calendar week. Even if we had seven consecutive days of sampling, extrapolation of findings to the whole of 2011 could not be justified, since we would have no idea whether those particular seven days were representative of the year. For example, holidays, parties or discovery of illicit drug production facilities could lead to bias. In Brussels, van Nuijs et al. (2011b) have, by analysing daily samples over a period of eight months, demonstrated significant differences in loads of DTRs including benzoylecgonine both between days and between months. More detailed study of temporal variation is clearly required before making generalisations outside of the sampling period.

We emphasise that what we are describing here are potential problems with the quality of some of the current data, rather than limitations of the statistical modelling approaches described. As we have discussed, bias might be reduced by obtaining better quality data, or by allowing multiple sources of data to inform a parameter. Alternatively, if the likely bias can be quantified using a distribution then this could be incorporated into the model (Eddy et al., 1992; Greenland, 2005). For example, by modelling more detailed data from elsewhere (e.g. van Nuijs et al., 2011b), it would be possible to define a distribution describing the likely size of bias associated with measuring samples over only a seven day period when what we are interested in is, say, a year. This distribution could then be incorporated into the statistical analysis. Similarly, the likely degree of a weekend peak in consumption might be quantified and incorporated, based either on published results or new modelling of other data sets.

## 6 Conclusions and discussion

We have proposed and demonstrated use of a Monte Carlo simulations approach to account for multiple sources of uncertainty when estimating illicit drug consumption using wastewater data. The approach produces credible intervals that more appropriately reflect the degree of uncertainty around estimates of drug consumption. To aid researchers in implementing this approach in future studies, we have provided web appendices demonstrating how this can be done. However, as the field develops further, it seems likely that extensions to the approach will increasingly be required. We have demonstrated two such extensions in Section 4, implemented within a Bayesian framework using MCMC simulation. We emphasise that this requires use of specialist software, and would highly recommend involvement of a statistician.

We have highlighted the importance of the distinction between standard deviations and standard errors. This distinction is crucial whatever approach is used to account for uncertainties. For example, consider the ‘RSD’ relating to excretion of cocaine as benzoylecgonine following nasal insufflation. From Table 2 we estimate this to be 0.02/0.316 = 6%. This is much smaller than the RSD of 25% reported by Castiglioni et al. (2013) for this parameter, on the basis of very similar data. This is because the latter incorporates inherent variability in excretion factors across individuals, which we have argued is not relevant to the estimation of average consumption.

The other crucial distinction we have made is that of uncertainty that is the result of statistical sampling error, and uncertainty due to systematic error (bias). We have made suggestions for how bias might be reduced or accounted for. However, it is important to realise that for some parameters the ideal is unlikely to ever be reached: for example, large scale pharmacokinetic studies across a range of routes of administration and doses, and in representative samples of drug users, seem unlikely due to the considerable costs and ethical issues.

In this paper we have focused on the ambitious target of accurately estimating absolute consumption of a drug by some population. In this context, all of the sources of uncertainty discussed are important. However, if instead examining trends over time (van Nuijs et al., 2011b) or across places (Banta-Green et al., 2009; Thomas et al., 2012) then some uncertainties appear less important. For example, Baker et al. (2014--in this issue) noted that 6-acetylmorphine lacks potential for estimation of absolute heroin usage, because of low and uncertain excretion rates. But if the aim of the research was to examine relative trends in heroin consumption over time in a stable population, then arguably this uncertainty might be of little relevance. Care would however be required to sample in a consistent manner, and to control for changes in the size and characteristics of the population. As we have noted, the size of the population served by an urban wastewater treatment plant is likely to vary even during a week. Average excretion factors might well also vary over a long period and across places, due to changes or differences in routes of administration or ethnic diversity.

An important research question is whether the information provided by wastewater analysis has the potential to contribute to estimates of the prevalence of illicit drug use. It has been noted previously that wastewater analysis is unlikely to replace more conventional approaches (Baker et al., 2014--in this issue; Frost et al., 2008). However, it is also well known that these conventional approaches lack a strong evidence base (Hickman et al., 2003). Population surveys can be unreliable even for commonly used drugs such as *Cannabis*, and will severely underestimate the use of rarer and more marginal drugs such as heroin, crack cocaine, and injecting behaviour. ‘Indirect’ estimation techniques such as capture–recapture or multiplier methods are therefore often used instead (e.g. Hickman et al., 2004; Kimber et al., 2008; King et al., 2009). However, these methods rely on untestable assumptions that might often be violated (Jones et al., in press) and different approaches can provide inconsistent estimates of prevalence (Singleton and Britain, 2006).

A Bayesian ‘multi-parameter evidence synthesis’ (MPES) framework (Ades et al., 2008; Hickman et al., 2013) would allow multiple sources of evidence on prevalence to be combined within a single model. The basic concept is the same as that described in Section 4: multiple sources of data are allowed to simultaneously inform particular parameters. Within such a model, information on drug use can also be related to information on relevant drug harms, such as drug-related mortality, so that information on both sides (prevalence and harm) can be used simultaneously to validate the other and generate coherent estimates (Hickman et al., 2013).

To incorporate wastewater data into such a model, the relationship between prevalence and consumption would need to be specified. A major challenge here is that there are several sub-populations of illicit drug users (recreational/dependent, mono/poly-drug users and, for cocaine, powder/crack cocaine users). Generally the focus of indirect methods such as capture–recapture is on estimating the number of problematic drug users, such as regular users of crack cocaine. For meaningful incorporation of the two types of data into a single model, estimates of the relative size of the different sub-populations, and of the average daily consumption of members of each group, would be required. Another aspect requiring careful consideration is the likely lack of alignment of geographical boundaries used across the different methods. It is uncertain whether these challenges could be adequately overcome. In addition, both types of analyses rely on a large number of assumptions with multiple uncertainties, and the flow of information around such networks of evidence is complex. Where an estimate depends on several uncertain parameters, reduction of uncertainty in part of the model has little impact on the final credible interval of interest unless it was the most uncertain parameter. This means that it is technically possible that incorporating wastewater data into such a model might lead to a reduction in uncertainty about, for example, routes of administration or average dosage, rather than about prevalence. As such, while there is little doubt that combining information from wastewater analysis and from epidemiological studies is computationally possible, it remains to be seen whether this would be worthwhile.

## Figures and Tables

**Table 1:** Daily measured flow volumes and concentrations of benzoylecgonine and norbenzoylecgonine, with standard errors (SEs). SD denotes the standard deviation of measured values.

		Mean	SE of mean	Source of information	Distribution assumed for parameter in back-calculations
Benzoylecgonine concentrations (ng per litre)	Day 1	1068.29	15.47	For each day the reported measured concentration is the mean over two samples. The SE is calculated as $\mathrm{SD}/\sqrt{2}$.	Normal (mean, SE^2^)
	Day 2	1054.89	22.89		
	Day 3	1167.18	24.91		
	Day 4	1544.00	27.96		
	Day 5	983.54	20.35		
	Day 6	1003.33	19.87		
	Day 7	907.00	6.65		
Norbenzoylecgonine concentrations (ng per litre)	Day 1	28.33	0.06		
	Day 2	33.59	0.69		
	Day 3	38.19	0.08		
	Day 4	53.95	0.64		
	Day 5	34.44	0.39		
	Day 6	31.08	0.57		
	Day 7	27.40	0.91		
Flow volume (millions of litres)	Day 1	1194.4	17.8	Flow was measured every 15 min and then averaged for each day. The SE is $\mathrm{SD}/\sqrt{n}$ where n = number of flow measurements in a day.	Normal (mean, SE^2^)
	Day 2	1178.0	16.4		
	Day 3	1180.0	15.6		
	Day 4	1172.4	20.7		
	Day 5	1158.6	22.2		
	Day 6	1161.7	18.0		
	Day 7	1155.5	17.0		

**Table 2:** Details of the distributions assumed for parameters. SD = standard deviation, SE = standard error. For beta distributions, a = ((1 − Estimate) / SE^2^ − 1 / Estimate) ×  Estimate^2^ and b = a ×  (1 / Estimate − 1).

Parameter	Estimate	SE of estimate	Source of information	Distribution assumed for parameter in back-calculations
*Population:* Size of population served by wastewater treatment plant (millions)	3.4	0.173	Population size estimate provided by water company personnel. The amount of uncertainty is unknown. Here we assume a 95% confidence interval around the estimate from 10% lower to 10% higher, implying that *SE* = 0.2 × *Estimate*/(2 × 1.96)	Normal (Estimate, SE^2^)
*ps:* Proportion of cocaine consumed by the population that smoked as free base	0.065	0.008	We examined the proportion of cocaine seized at police stations that was crack cocaine, over the last five years (Coleman, 2012). We used the mean of these five proportions and the SE of this mean.	Beta (a, b)
*es_1_:* Proportion of a dose of cocaine smoked as free base that is excreted as benzoylecgonine	0.087	0.009	We performed a fixed effect meta-analysis of data presented in Khan and Nicell (2011) from three small pharmacokinetic studies.	Beta (a, b)
*es_2_:* Proportion of a dose of cocaine smoked as free base that is excreted as norbenzoylecgonine	0.002	0.001	Data presented in Khan and Nicell (2011) from a single small pharmacokinetic study (Cone et al., 1998).	Beta (a, b)
*en_1_:* Proportion of dose of cocaine taken by nasal insufflation that is excreted as benzoylecgonine	0.316	0.020	We performed a random effects meta-analysis of data presented in Khan and Nicell (2011) from six small pharmacokinetic studies.	Beta (a, b)
*en_2_:* Proportion of dose of cocaine taken by nasal insufflation that is excreted as norbenzoylecgonine	0.010	0.002	Data presented in Khan and Nicell (2011) from a single small pharmacokinetic study (Cone et al., 1998).	Beta (a, b)
*Stability_1_:* Percentage increase in concentration of benzoylecgonine in unfiltered wastewater	5.5	2.1	Values from Baker and Kasprzyk-Hordern (2011a) for pH 7.4, 19 °C, 12 h. These were averages across two samples, hence $\mathrm{SE}=\mathrm{SD}/\sqrt{2}$	Normal (Estimate, SE^2^)
*Stability_2_:* Percentage increase in concentration of norbenzoylecgonine in unfiltered wastewater	3.5	4.3		

## Appendix A Supplementary data

Supplementary material 1. Excel spreadsheet demonstrating propagation of uncertainty using Monte Carlo simulation.

Supplementary material 2. Code for the free statistical software R, demonstrating propagation of uncertainty using Monte Carlo simulation.
